# The mechanism of body–mind integration in the formation of destination attachment: A comparison of first-time and repeat tourists

**DOI:** 10.3389/fpsyg.2022.1010589

**Published:** 2022-11-10

**Authors:** Yinyin Dong, Ying Qu

**Affiliations:** ^1^Tourism College, Hainan University, Haikou, Hainan, China; ^2^School of Tourism and Urban-Rural Planning, Zhejiang Gongshang University, Hangzhou, Zhejiang, China

**Keywords:** multisensory impressions, embodied theory, dual-system theory, emotional responses, cognitive responses, destination attachment

## Abstract

By constructing a person-body–mind-place framework of destination attachment, this study explores the physical and mental formation mechanism of destination attachment and examines its dynamics between first-time and repeat tourists. The present study found that multisensory impressions can, directly and indirectly, affect destination attachment through emotional and cognitive psychological mediation. There are differences between first-time tourists and repeat tourists in terms of this mediation path. As the frequency of travel increases, the influence of multisensory impressions gradually increases. The formation of destination attachment is dominated by emotion for first-time tourists, whereas it is dominated by cognition for repeat tourists. Based on these findings, theoretical and practical implications are presented.

## Introduction

As a representation of the human-land relationship, destination attachment has unique tourism marketing value (Ramkissoon, 2016), and exploring its formation mechanism is of great significance to tourism industry. However, previous studies tend to explain the formation of destination attachment in terms of either social construction (i.e., the perspective of the body) or subjective construction (i.e., the perspective of the mind) while ignoring the process of body–mind unity in the formation of destination attachment (Kastenholz et al., 2020). With the increasing rise of embodied theory emphasizing the “oneness of mind and body” in tourism research, its explanatory power for destination attachment has been recognized by some scholars (Yuksel et al., 2010). However, systematic integration studies have yet to be conducted.

Destination attachment is formed through the interaction of tourists with the tangible/intangible environment of the destination, and it is embodied in the process of travel experience (Loureiro, 2014). Tourists use their five senses to get information about a destination. Multisensory impressions are the first impression of tourists after entering the destination, which directly determines the perception, attitude, and behavior of tourists during the tour (Manosuthi et al., 2021). These impressions naturally become a key factor affecting destination attachment (Lv and McCabe, 2020). In addition, when tourists’ bodies are in a tourist situation, their psychological states may be activated (Krishna, 2012). Therefore, the interaction between tourists and the destination involves multisensory stimulation and emotional and cognitive psychological processes (Rakić and Chambers, 2012). Emotion and cognition coexist in attachment relationships as potential components of destination attachment (Scannell and Gifford, 2010). Although previous studies have recognized the importance of individual psychological factors on destination attachment (Prayag and Lee, 2019), they have not differentiated between emotional and cognitive pathways in shaping the different dimensions of destination attachment.

Moreover, due to their different travel experiences, first-time tourists may be more likely to trigger emotional reactions after receiving information through the senses. In contrast, with increased travel frequency, repeat tourists emphasize psychological meaning (Fuchs and Reichel, 2011). While previous studies have confirmed that a difference exists in the intensity of destination attachment between first-time and repeat tourists (Morais and Lin, 2010), determining how and in which ways first-time and repeat tourists diverge in the psychological mechanism of sensory impression influencing destination attachment has yet to be examined.

Therefore, this study aims to explore the relationship between multisensory impressions, emotional responses, cognitive responses, and destination attachment and identify differences in these relationships between first-time and repeat tourists. The shortcomings of existing research will be accounted for by achieving the above goals, and the mind–body mechanisms underlying the dynamic formation of destination attachment will be discovered.

## Literature review and hypotheses development

### Destination attachment and its formation mechanism

Rooted in the attachment theory, destination attachment is defined as the cognitive and emotional linkage that tourists establish with a tourism destination (Japutra, 2020), reflecting the extent to which an individual values and identifies with a particular environmental setting (Yuksel et al., 2010). The sense of physically being and feeling “at home” can be considered a sign that a tourist creates a connection to the destination. Although destination attachment has been mentioned as a multidimensional construct (Io and Wan, 2018), the two-dimensional division, which includes place identity and place dependence (Yuksel et al., 2010), is widely recognized by scholars (Liu et al., 2019).

Place identity refers to a tourist’s rich memories and affection for a particular destination, which are preserved in the definition of self. Place identity underscores the cognitive domain of a sense of place, which is related to symbolic meanings that a tourist ascribes to and self-identifies with that destination (Williams and Vaske, 2003). Place identity can be developed through positively balanced perceptions (Dwyer et al., 2019). Place dependence refers to the collection of social and material resources that meet the specific needs of tourists and represent the unique qualities of a place. Place dependence is a form of functional attachment, providing features and conditions that support achieving specific goals or desired activities (Williams and Vaske, 2003). Place dependence occurs when tourists show a functional need for a destination that is not transferable to another destination (Mlozi and Pesämaa, 2013). In the existing research on the measurement of the destination attachment model, place dependence and place identity are often used as second-order latent variables to conduct path tests (Hosany et al., 2017; Kastenholz et al., 2020). Although this can encompass the overall characteristics of destination attachment from a macro perspective, it ignores the complexity of the internal structure of destination attachment and the uniqueness of the formation mechanism of different dimensions (Mlozi et al., 2012). Therefore, the importance of refining the two-dimensional construct of destination attachment has been well approved (Mlozi et al., 2012).

Developing a market base made up of attached tourists is important, as they are less likely to change their choice of place despite the offerings of the alternatives (Loureiro, 2014). Destination attachment has the potential to improve satisfaction (Ramkissoon, 2016), foster attitudinal loyalty (Yi et al., 2018), expand word-of-mouth advocacy (Pandey and Sahu, 2020), and promote revisiting (Jian et al., 2021). Destination attachment is even seen as the key to enhancing the competitiveness of tourist destinations through increased patronage and profits (Dwyer et al., 2019). Exploring the formation mechanism of purposive attachment thus becomes an important topic.

There are two main paradigms of place attachment formation mechanisms. The earliest is social constructivism, which emphasizes physical practice and gives spatial meaning through interaction (Tuan, 1975). An abstract space becomes a meaningful place through experience (Kastenholz et al., 2020). Individuals are not directly attached to places but to the symbolism they represent. The classic “place ballet” view explains how people’s movement in space and time forms the process of place meaning (Seamon, 1980). However, this physically-based idea of the formation of place attachment is limited to theoretical discussion and lacks empirical evidence. Furthermore, the single view of physical practice ignores the complex psychological activities of tourists in the tourism situation.

The more recent subjective constructivism synthetically describes the formation of place attachment by a tripartite framework of “people-place-psychological processes” (Scannell and Gifford, 2010). Psychological processes link people with a place, thus dominating this research branch. However, existing research explores the effect of a comprehensive psychological variable that blends the cognitive and affective effects (e.g., motivations, satisfaction, personal involvement, destination image, perceived attractiveness, and self-congruity) on place attachment as a one-dimensional concept (Prayag and Ryan, 2012; Xu and Zhang, 2016; Riper et al., 2019; Tasci et al., 2022; Usakli et al., 2022). Little is known about the concrete effect of cognition and emotion in isolation on place attachment, especially on the different dimensions of place attachment. Moreover, this research branch also ignores the influence of physical elements in co-shaping place attachment. Therefore, the research gaps are two-fold. First, on the whole, the process of body–mind unity in forming destination attachment has yet to be studied. Second, regarding the mind branch, the separation effect of cognition and emotion on the different dimensions of destination attachment has to be delineated. The two gaps will be bridged together in this study.

### Embodied theory and multisensory impressions

The embodied theory emphasizes that consumers’ physical senses first receive external marketing stimuli, which is then processed by consumers’ psychology, followed by affecting their attitudes and behaviors (Walther-Hansen, 2020). The senses are not independent of psychology but rather participate in the process of psychology. The mind must be understood in the context of its relationship to the body, which is the process of “body–mind oneness”. When the external environment stimulates different sensory cells of tourists, tourists first form different “sensations” and then produce a “conscious sensory experience” (Ji and King, 2018). In the research topic of the people-land relationship under the embodied paradigm, the body of tourists is similar to the plasma membrane of cells, which plays a role in material exchange and energy transfer (Vaske and Kobrin, 2001). In this connection between the inside and outside of the interface, the birth of a new people-land relationship occurs.

The concept of multisensory impressions first appeared in the field of sensory marketing and has recently attracted the interest of tourism scholars (Manosuthi et al., 2021). Fakfare et al. (2021) found that multisensory impressions are a good way to determine the perception of tourists’ degree of sensory stimulation when visiting a destination and can be summarized into five aspects: sight, hearing, taste, touch, and smell (Chen et al., 2021). Agapito et al. (2017) posited that multisensory impressions result from screening stimuli and experiences by tourists’ bodily senses. Usually, only those unique, profound, and valuable sensory stimuli and experiences will leave multisensory impressions. In recent years, multisensory experiences have been mentioned as an important prerequisite for destination attachment formation. Human geography studies have shown that the five senses are crucial for developing human-land relationships (Xiong et al., 2015). Tourists interact with destinations through their five senses (Agapito et al., 2017), stimulating an emotional preference for destinations. Multisensory impressions reflect the quality of the travel experience and link the objective physical environment with subjective emotional attachment (Agapito et al., 2014). Extraordinary multisensory impressions not only enhance tourists’ intimacy with the destination but may also trigger more identification with the destination (Lv and Wu, 2021). Multisensory impressions satisfy tourists’ needs for sensory pleasure and prompt tourists to reflect on the relationship between themselves and the destination, which is conducive to the formation of place dependence and place identity (Yang et al., 2021). The stronger the multisensory impression, the stronger the visitor’s attachment to the destination is likely to be. Therefore, this study proposes the following:

*H1*: Multisensory impressions have a significant positive effect on destination attachment.

*H1a*: Multisensory impressions have a significant positive impact on place dependence.

*H1b*: Multisensory impressions have a significant positive impact on place identity.

### “Emotion-cognition” psychological systems

After external stimuli are input into the body of tourists through the senses, the tourists still need to go through a complex psychological process before they can have a conscious sensory experience, affecting subsequent behavioral choices (Jiang, 2020). Some scholars have proposed the “emotion-cognitive dual system model” to subdivide the complex psychological state of individuals (Alyahya and McLean, 2021). The dual system theory is also regarded as an information processing theory, which explains the formation of individual attitude preferences through two different information processing pathways (the limbic pathway of the emotional system and the central pathway of the cognitive system; Alyahya and McLean, 2021). Among them, the emotional system adopts the principle of intuition, requiring individuals to process information quickly with less effort. In contrast, the cognitive system adopts the principle of rationality, requiring individuals to use enough cognitive resources to comprehensively evaluate the content of the information. Emotional and cognitive responses trigger travel destination preferences (Michael et al., 2019).

Emotions represent distinct mental states characterized by episodes of intense feelings associated with a specific referent and instigate specific response behaviors, which are often unconsciously aroused. Emotions fundamentally shape the tourism experience; particularly, positive emotions are the core of hedonic tourism (Kim and Fesenmaier, 2015). Empirical evidence highlights the outstanding role of positive emotions in tourism, positively arousing tourist experiences connected to increased satisfaction, memorability, and loyalty (Agapito et al., 2017). Hosany and Gilbert (2010) developed the Destination Emotion Scale, arguing that positive emotion in tourism destinations involves a psychological state characterized by joy, love, and positive surprise.

However, tourists go beyond the superficial reception of sensory information and enter the consciousness domain of the mind (Wen and Leung, 2021), triggering deep-level cognition such as imagination, association, and thinking (Collins and Allard, 2001). Cognitive responses are the collection of all mental abilities and processes related to knowledge, memory, judgment, and even decision-making, which is a conscious mental process (Brown and Raymond, 2007). Tourists’ thinking and understanding of the destination environment occur throughout the entire tourism process. There is widespread support for cognitive assessment of destinations regarding goal congruence, certainty, and novelty (Choi and Choi, 2019).

Although emotion and cognition are independent psychological processes, they are closely related (Plass and Kalyuga, 2019). The broaden-and-build theory of positive emotions suggests that positive emotions promote cognitive processing (Gable and Harmon-Jones, 2010). The emotions evoked by tourists interacting with the destination environment are all prerequisites for evaluating the cognitive experience of tourism (Tsai et al., 2020). Therefore, this study proposes the following:

*H2*: Emotional responses have a significant positive impact on cognitive responses.

Sensory marketing theory emphasizes that consumers interact with the outside world through their senses, affecting their emotions, attitudes, memories, and behaviors (Sthapit, 2019). Sensory stimuli, which are regarded as key tools for creating a tourism experience, are an important prerequisite for activating tourists’ emotional and cognitive responses. Tourists’ multisensory impressions partly explain the positive emotions associated with travel experiences (Yang et al., 2021). In the context of rural tourism, Kastenholz et al. (2020) verified the positive effects of multisensory impressions on emotions, and different sensory impressions have different effects on different dimensions of emotion. Lindstrom (2005) found that tactile experience stimulated tourists’ positive emotions and played a significant role in decision-making. Furthermore, tourists’ cognitive process of a destination is also deeply rooted in the body’s interaction with the world (Barsalou, 2008). Embodied tourism activities are also of great value in enhancing tourists’ perceptions (Walther-Hansen, 2020). Multisensory impressions can stimulate the perception and imagination of tourists, resulting in a more profound cognitive effect (Liu et al., 2019). Rich multisensory impressions play a crucial role in promoting tourists’ cognitive memory of a destination (Agapito et al., 2017). The stronger the sensory impression, the stronger the emotional and cognitive responses of visitors. Therefore, this study proposes the following hypotheses:

*H3*: Multisensory impressions have a significant positive impact on emotional responses.

*H4*: Multisensory impressions have a significant positive impact on cognitive responses.

Destination attachment is generally multifaceted, involving at least the cognitive interpretations of emotional responses to environmental stimuli relating to a particular geographical area (Hidalgo and Hernandez, 2001). Therefore, the formation of destination attachment involves both an emotional path and a cognitive path. First, attachment is regarded as an adaptive emotional response of an individual in a specific social relationship in psychology. Positive emotions are essential for building attachment to a destination (Grisaffe and Nguyen, 2011). High levels of positive emotions lead to strong destination attachment (Yan and Halpenny, 2019). Kim and Fesenmaier (2015) argued that positive emotions could activate on-site peak experiences and affect the recall of attachment memories. Fredrickson (2001) believed that tourists who receive more positive emotions through tourism experience might participate in more travel activities, promoting the development of place dependence. Io (2018) found that positive emotions promote tourist satisfaction and trigger tourists’ thinking about destination identity. Second, although a person’s attachment orientation is often conceptualized as a single global orientation toward close relationships, it is rooted in a complex network of cognitive processes. Current studies argue that a person’s cognitive evaluation of the experience is a necessary and sufficient condition for attachment to be formed (Gillath et al., 2009). Tourists’ cognitive assessments of destination travel experiences may alter a person’s attachment orientation. For example, the higher the perceived value of the landscape, the stronger the dependence on the destination (Brown and Raymond, 2007). When tourists associate their travel goals with themselves, they tend to identify with the destination (Barsalou, 2008). Therefore, this study proposes the following:

*H5*: Emotional responses have a significant positive impact on destination attachment.

*H5a*: Emotional responses have a significant positive impact on place dependence.

*H5b*: Emotional responses have a significant positive impact on place identity.

*H6*: Cognitive responses have a significant positive impact on destination attachment.

*H6a*: Cognitive responses have a significant positive impact on place dependence.

*H6b*: Cognitive responses have a significant positive impact on place identity.

### Path difference between first-time and repeat tourists

It has been widely confirmed that there are differences in travel behaviors (e.g., motivation, experience, satisfaction, and revisit intention) between first-time and repeat tourists (Shanka and Taylor, 2004; Morais and Lin, 2010; Hsu et al., 2014). As the value of destination attachment becomes more pronounced, studies have begun to explore differences in destination attachment between first-time and repeat tourists. For example, Lewicka (2011) believed that tourists’ attachment to a destination gradually increases with the increase in travel frequency. Similarly, Vada et al. (2019) found that tourists more familiar with a destination are more likely to have a close relationship with the destination. Morais and Lin (2010) found that first-time tourists were mainly influenced by destination image, while repeat tourists were influenced by place attachment. Although these studies focused on the difference in the manifestation and effect of destination attachment, the differences in physical and mental mechanisms in the formation of destination attachment between first-time and repeat tourists have been ignored. Destination attachment is a product of the interaction of tourists with the destination. Tourists with different travel experiences have different degrees of received destination information through their senses, leading to differences in psychological activities and affecting the relationship between tourists and the destination.

For first-time tourists, due to the lack of prior experience with the destination, the landscape stimuli of the destination are a new experience for them, which may trigger intuitive emotional responses and do not require a lot of cognitive resources (Fuchs and Reichel, 2011). In addition, due to the short contact time with the destination, first-time tourists generally form a superficial attachment to the destination. Repeat tourists have a certain degree of knowledge about the destination (Shanka and Taylor, 2004). When repeat tourists are immersed in the scene again, the tourists will fully mobilize their cognitive resources to process the information received by the senses (Hwang et al., 2005). Generally speaking, the revisit is given more meaning, a process of spiritual awakening and self-reflection for tourists. Furthermore, with the increase in the frequency of travel, tourists’ attachment to the destination is more reflected in symbolic attachment. Accordingly, this study proposes the following:

*H7*: There are significant differences in the relational pathways of multisensory impressions, emotional responses, cognitive responses, and destination attachment between first-time tourists and repeat tourists.

## Study design

### Case study context

As the southernmost and only tropical coastal destination in China, Hainan Island has a long history with the development of coastal vacation tourism. However, in recent years, the characteristics of this destination entering a mature/stagnant period have become more prominent. Since 2012, the growth rate of domestic tourists in Hainan has been relatively low (10%), showing a steady trend with alternating slight declines and low recovery. Sanya, its core attraction area, has seen a more pronounced decline. The traditional functional marketing methods of Hainan destinations, which mainly focus on promoting destination attributes, are slightly exhausted. In contrast, relationship marketing methods emphasizing establishing an affective connection with the destination may provide an opportunity to reverse the unfavorable situation. Therefore, by taking Hainan Island as a case study, the “body–mind utility” shaping mechanism of destination attachment is explored.

### Measures

The research questionnaire is divided into two parts. The first part is the central part of the questionnaire, including the scales of different variables, while the second part is the personal information of tourists. The measurement items of each variable in the model are from mature scales widely used in the relevant literature. The English scale has been appropriately modified according to the specific situation of the Hainan destination. The survey was first developed in English and then translated into Chinese by accredited translators. It was later translated back to English to ensure that the meanings of the survey items did not get lost during the process.

Among them, multisensory impressions refer to the research of Lv and McCabe (2020), Santos et al. (2019), and Fakfare et al. (2021), which covers the five dimensions of visual impression, auditory impression, gustatory impression, olfactory impression, and tactile impression. Emotional responses refer to research on destination emotion conducted by Hosany and Gilbert (2010), Hosany et al. (2015), and Hosany and Prayag (2013). The three dimensions of joy, love, and positive surprise were selected. Cognitive responses refer to the research conducted by Ma et al. (2019), Rivera et al. (2019), and Zheng et al. (2019) on cognitive assessment in tourism contexts. The three dimensions of goal consistency, certainty, and novelty were selected. Destination attachment refers to the research of Prayag and Lee (2019) and Liu et al. (2019), which includes the two dimensions of place dependence and place identity. All variables were measured using a 5-point Likert scale (1 = completely disagree, 5 = completely agree).

Before the formal investigation, this study conducted a pre-test to verify the reliability and validity of the scale. The pre-test was conducted in Sanya Bay, one of Hainan’s most famous and tourist-concentrated scenic spots. A total of 100 questionnaires were distributed through systematic random sampling, and 86 valid questionnaires were recovered. The pre-test results show that the Cronbach’s alpha coefficient of each construct is greater than 0.7, indicating that the scale has good reliability; the factor loading of each item is above 0.7, indicating that the scale has good construct validity (Hair et al., 2021).

### Data collection

Given the particularity of the formation of destination attachment (such as requiring a certain length of human-land interaction), by referring to the practice of previous research (e.g., Prayag and Lee, 2019), the present study excludes non-overnight excursionists who stay for less than 1 day and chooses the formal research time from October 5th to October 7th, 2021. This period is when Hainan tourism begins to enter its peak season, and China’s National Day holiday is longer, making it easy for tourists to immerse themselves in the tour. In such a context, the sample quality is high, thereby improving the validity of the sampling. In addition, to make the sample representative, the Sanya Phoenix Airport and Haikou Meilan Airport in Hainan Province (the leading import and export channels for Hainan tourism) were selected as the questionnaire distribution places, and the survey was mainly conducted by a random interception with an interval of every five tourists. Furthermore, to ensure the quality of the questionnaire collection, the questionnaire distributors consisted of postgraduates majoring in tourism management (7 students in total), who were more qualified for survey management.

First, the respondents were asked whether they planned to leave the island after traveling in Hainan. In this way, local residents, transfer passengers, and tourists who have just entered the island were excluded from the study. After getting a confirmed answer, the questionnaire was shown to the tourists. Due to the lengthy questionnaire questions, a small gift was given to express gratitude to the tourists. A total of 700 questionnaires were distributed during the period. Excluding those filled incompletely, 670 valid samples were recovered, and the effective recovery rate was 95.71%. The questionnaires split between first-time tourists and repeat tourists amounted to 332 and 338, respectively. Generally, the number of samples should be at least 10 times the number of variables (Hair et al., 2021). In addition, the G*Power program was used to calculate the sample size required, based on an effect of 0.15 for the predictors, a precision level of 5%, a test power of 0.97 and 32 predictors (Memon et al., 2020). The sample size required was 291 respondents. Thus, the current sample size (more than 300 for each group) was sufficient.

The sample was almost evenly divided between males and females for both groups. Most respondents were between the ages of 18–40 (first-timers: 82.8%; repeat tourists: 65.1%) and held a university degree (first-timers: 76.1%; repeat tourists: 76.2%). The majority of respondents were full-time employed professionals (first-timers: 50.5%; repeat tourists: 49.5%).

### Data analysis

This study used partial least squares-structural equation modeling (PLS-SEM) for analysis. The reasons are as follows. Firstly, PLS provides a variance-based predictive SEM method with the ability to analyze complex models and simultaneously handle reflective, formative, and higher-order model structures (Henseler et al., 2015). Secondly, PLS does not have data restrictions regarding normal distribution, randomness, and sample size, and the requirements are relatively loose, thereby providing flexibility for data analysis (Hair et al., 2021). Thirdly, PLS can effectively manage interfering data and missing values with good predictive and explanatory power.

## Results

### Measurement model

As shown in Tables 1, 2, firstly, the combined reliability (CR) and Cronbach’s alpha coefficient of each variable were greater than 0.7 in both the first-time and repeat groups, indicating that all scales had good reliability (Bagozzi and Yi, 1988). Secondly, the factor loadings of all items and the average variance extraction (AVE) in both groups were higher than the threshold of 0.5, indicating that the model had good convergent validity (Fornell and Larcker, 1981). Furthermore, for both first-time and repeat tourists, the values of the heterotrait-monotrait (HTMT) were all below 0.85, indicating that each variable had good discriminant validity (Hair et al., 2021). In addition, the variance inflation factor (VIF) value ranged from 2.124 to 3.581 for both groups, which was less than the threshold of 5.000, indicating that multicolinearity was not an issue in this research (Hair et al., 2021).

**Table 1:** Assessment results of the measurement model.

Items	Statements	Loading		Cronbach’s Alpha		CR		AVE	
		First	Repeat	First	Repeat	First	Repeat	First	Repeat
Visual impression	Ocean landscape	0.916	0.891	0.846	0.870	0.907	0.911	0.766	0.812
	Natural astronomical	0.844	0.920						
	Historical buildings	0.861	0.781						
Auditory impression	Bird sounds	0.907	0.830	0.852	0.854	0.931	0.913	0.871	0.760
	Dialect	0.810	0.791						
Gustatory impression	Seafood	0.947	0.856	0.863	0.803	0.935	0.890	0.879	0.753
	Tropical fruits	0.842	0.867						
Olfactory impression	Fresh air	0.918	0.907	0.823	0.819	0.918	0.912	0.849	0.871
	Floral fragrance	0.859	0.896						
Tactile impression	Water sports	0.937	0.931	0.821	0.854	0.917	0.906	0.847	0.834
	Soft feeling	0.903	0.887						
Joy	Enthusiasm	0.905	0.912	0.835	0.801	0.901	0.911	0.753	0.811
	Delight	0.857	0.798						
Love	Caring	0.894	0.906	0.841	0.835	0.904	0.932	0.759	0.781
	Affection	0.856	0.912						
	Tenderness	0.862	0.790						
Positive surprise	Amazement	0.913	0.913	0.843	0.825	0.906	0.900	0.762	0.766
	Fascinated	0.834	0.790						
Goal congruence	Achieving your needs	0.926	0.912	0.881	0.813	0.927	0.911	0.808	0.723
	Achieving your desires	0.878	0.897						
	Achieving life goals	0.892	0.900						
Certainty	You understand what was happening	0.952	0.914	0.878	0.815	0.942	0.936	0.891	0.819
	You are sure of what was happening	0.936	0.840						
Novelty	Unexpected	0.912	0.923	0.814	0.800	0.89	0.912	0.73	0.88
	Unusual	0.832	0.815						
	Unfamiliar	0.816	0.890						
Place dependence	Hainan is the best place	0.936	0.876	0.900	0.810	0.926	0.912	0.715	0.890
	I get more satisfaction from Hainan	0.829	0.912						
	Hainan is more important to me	0.836	0.826						
Place identity	Hainan means a lot to me	0.809	0.837	0.871	0.839	0.912	0.923	0.722	0.790
	I identify strongly with Hainan	0.838	0.901						
	I have become part of Hainan	0.890	0.923						

**Table 2:** HTMT discriminant validity analysis results (first-time tourists/repeat tourists).

Sequence	Item	1	2	3	4	5	6	7	8	9	10	11	12	13
1	Visual impression													
2	Auditory impression	0.574/0.641												
3	Taste impression	0.556/0.447	0.520/0.432											
4	Olfactory impression	0.499/0.371	0.672/0.312	0.662/0.551										
5	Tactile impression	0.451/0.301	0.456/0.380	0.543/0.541	0.441/0.446									
6	Joy	0.491/0.502	0.447 0.302	0.559/0.500	0.565/0.309	0.458/0.231								
7	Love	0.683/0.544	0.546/0.456	0.444/0.308	0.481/0.377	0.458/0.543	0.672/0.500							
8	Positive surprise	0.597/0.470	0.474/0.543	0.372/0.411	0.592/0.444	0.595/0.431	0.597/0.501	0.508/0.602						
9	Goal congruence	0.397/0.421	0.453/0.324	0.609/0.599	0.670/0.625	0.649/0.570	0.565/0.311	0.373/0.441	0.411/0.605					
10	Certainty	0.507/0.556	0.661/0.602	0.730/0.423	0.688/0.570	0.754/0.690	0.476/0.280	0.683/0.423	0.502/ 0.200	0.682/0.443				
11	Novelty	0.685/0.466	0.746/0.701	0.594/0.610	0.787/0.702	0.665/0,234	0.487/0.462	0.775/0.651	0.507/0.552	0.791/0.534	0.580/0.551			
12	Place dependence	0.504/0.511	0.554/0.543	0.556/0.552	0.383/0.367	0.485/0.467	0.683/0.602	0.384/0.301	0.602/0.612	0.569/0.345	0.772/0.678	0.380/0.225		
13	Place identity	0.590/0.432	0.642/0.432	0.671/0.603	0.387/0.467	0.474/0.511	0.486/0.467	0.488/0.543	0.396/0.400	0.478/0.287	0.393/0.443	0.593/0.430	0.501/0.430	

### Structural model

As shown in Table 3, the *R*^2^ results of both groups (first-timers: between 38.1 and 78.9%; repeat tourists: between 55.2 and 64.9%) showed that the predictive ability of the model constructs reached a medium level or above (Hair et al., 2021). The *Q*^2^ values were all greater than 0 (first-timers: between 0.271 and 1.817; repeat tourists: between 0.502 and 1.752), indicating that the exogenous constructs had a certain predictive ability to endogenous constructs. The effect size (*f*^2^) values of the proposed structural model were all higher than the standard value of 0.02 for both groups (Hair et al., 2021). The standardized root mean square residual (SRMR) values of the model in the two groups were 0.065 and 0.061, respectively, which met the criterion of less than 0.08 proposed by Henseler et al. (2015), indicating that the model had a good model fit. The acceptability and invariance of the measurement model were also confirmed.

**Table 3:** Structural model test results.

	R^2^		Q^2^		The effect size (*f* ^2^)							
					Emotional responses		Cognitive responses		Place dependence		Place identity	
	First	Repeat	First	Repeat	First	Repeat	First	Repeat	First	Repeat	First	Repeat
Multisensory impressions					0.632	0.582	0.373	0.412	0.146	0.161	0.135	0.154
Emotional responses	71.9%	64.9%	1.817	1.752			0.263	0.234	0.067	0.108	0.077	0.046
Cognitive responses	63.1%	65.1%	1.091	1.544					0.031	0.041	0.049	0.059
Place dependence	48.4%	55.2%	0.360	0.502								
Place identity	38.1%	57.1%	0.271	0.521								

### Direct effects test and multi-group analysis

**Figure 1:**
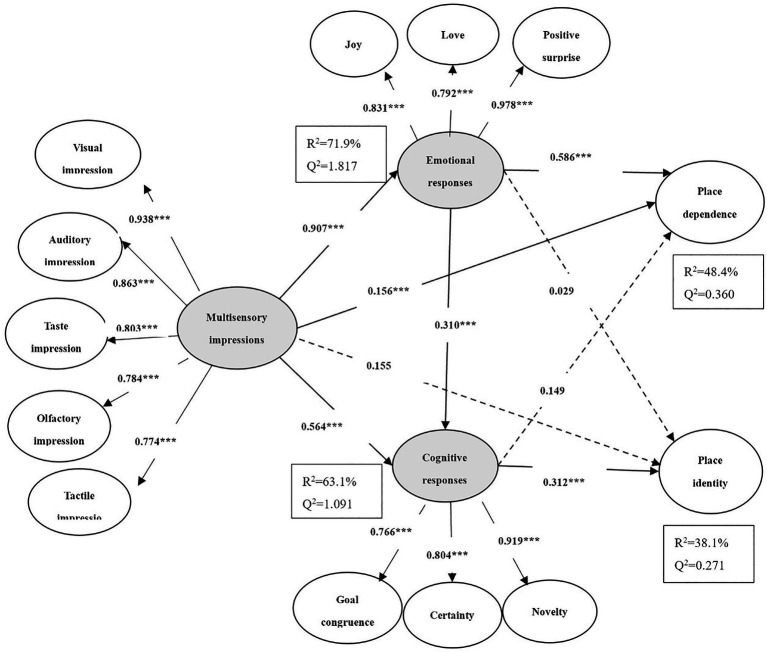
Conceptual model for first-time tourists. * indicates significance at *p* < 0.05; ** indicates significance at *p* < 0.01; and *** indicates significance at *p* < 0.001.

**Figure 2:**
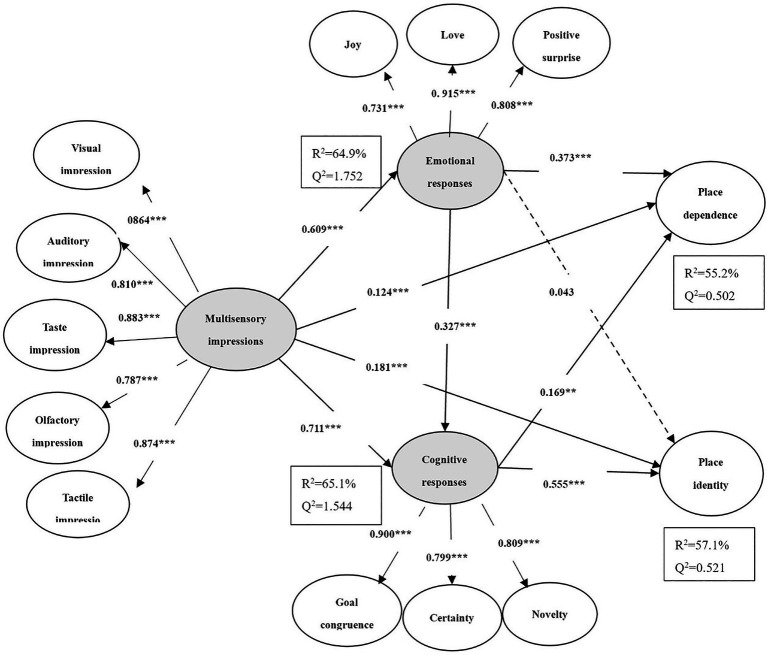
Conceptual model for repeat tourists. * indicates significance at *p* < 0.05; ** indicates significance at *p* < 0.01; and *** indicates significance at *p* < 0.001.

First, the invariance of the two groups was assessed using the measurement invariance of composite models (MICOM) procedure of PLS-SEM, which showed that configural invariance, compositional invariance, equal mean values, and variances were all well-established (Hair et al., 2021). Hypothesis testing and multi-group analysis results can therefore be carried out. The test results are shown in Figures 1, 2, as well as in Table 4.

**Table 4:** Hypothetical test and multi-group analysis.

Path relationship	Path coefficients		Path coefficient differences	*p*-value Henseler’s MGA
	Original (first-time)	Original (repeat)		
Multisensory Impressions→Place Dependence	0.156***	0.124***	0.032	0.594
Multisensory Impressions→Place Identity	0.155	0.181***	−0.026***	0.000
Emotional Responses→Cognitive Responses	0.310***	0.327***	−0.017	0.559
Multisensory Impressions→Emotional Responses	0.907***	0.609***	0.298*	0.012
Multisensory Impressions→Cognitive Responses	0.564***	0.711***	−0.147*	0.038
Emotional Responses→Place Dependence	0.586***	0.373***	0.213***	0.000
Emotional Responses→Place Identity	0.029	0.043	−0.014	0.803
Cognitive Responses→Place Dependence	0.149	0.169**	0.020**	0.006
Cognitive Responses→Place Identity	0.312***	0.555***	−0.243*	0.046
Multisensory Impressions→Emotional Responses→Place Dependence	0.474***	0.386***	0.088***	0.000
Multisensory Impressions→Emotional Responses→Place Identity	0.017	0.296	−0.279	0.505
Multisensory Impressions→Cognitive Responses→Place Dependence	0.037	0.015**	0.022*	0.02
Multisensory Impressions→Cognitive Responses→Place Identity	0.313***	0.451***	−0.138***	0.000

* indicates significance at *p* < 0.05; ** indicates significance at *p* < 0.01; and *** indicates significance at *p* < 0.001.

The results show that multisensory impressions significantly and positively affected place dependence (first-timers: *β* = 0.156; *p* < 0.001; repeat tourists: *β* = 0.124; *p < 0.*001) across the two groups, H1a was thus supported. However, there was no significant difference between first-time tourists and repeat tourists for hypothesis H1a (*β_first_-β_repeat_* = 0.032, *p > 0.05*). As for the relationship between multisensory impressions and place identity, H1b was partially supported across the two groups because the positive relationship existed not for first-timers but for repeat tourists (first-timers: *β* = 0.155, *p* > 0.05; repeat tourists: *β* = 0.181, *p* < 0.001). Moreover, the difference that multisensory impressions had on place identity between the two groups was significant (*β_first_-β_repeat_* = −0.026, *p* < 0.001). Hypothesis H2 was also supported across the two groups, showing a significant relationship between emotional responses and cognitive responses (first-timers: *β* = 0.310, *p* < 0.001; repeat tourists: *β* = 0.327, *p* < 0.001). However, hypothesis H2 showed no significant difference between first-time tourists and repeat tourists (*β*_first_-*β*_repeat_ = −0.017, *p* > 0.05).

In addition, multisensory impressions were found to be positively related to emotional responses (first-timers: *β* = 0.907, *p* < 0.001; repeat tourists: *β* = 0.609, *p* < 0.001) and cognitive responses (first-timers: *β* = 0.564, *p* < 0.001; repeat tourists: *β* = 0.711, *p* < 0.001) across the two groups, thus supporting hypotheses H3 and H4. For both groups, multisensory impressions had a significant difference in emotional responses (*β_first_-β_repeat_* = 0.298, *p* < *0.05*) and cognitive responses (*β_first_-β_repeat_* = −0.147, *p* < *0.05*). Emotional responses significantly affected place dependence for the two groups (first-timers: *β* = 0.586, *p* < 0.001; repeat tourists: *β* = 0.373, *p* < 0.001). However, emotional response did not significantly affect place identity for the two groups (first-timers: *β* = 0.029, *p* > 0.05; repeat tourists: *β* = 0.043, *p* > 0.05). Thus, hypothesis H5a was accepted, while H5b was rejected. For both groups, emotional responses had a significant difference in place dependence (*β_first_-β_repeat_* = 0.213, *p* < 0.001), but not in place identity (*β_first_-β_repeat_* = −0.147, *p* > 0.05). Similarly, cognitive response significantly affected place dependence for repeat tourists, but not for first-timers (first-timers: *β* = 0.149, *p* > 0.05; repeat tourists: *β* = 0.169, *p* < 0.01). However, cognitive response significantly affected place identity for the two groups (first-timers: *β* = 0.312, *p* < 0.001; repeat tourists: *β* = 0.555, *p* < 0.001). Hypothesis H6a was partially supported, and H6b was accepted. Nevertheless, cognitive responses had a significant difference in place dependence (*β_first_-β_repeat_* = 0.020, *p* < 0.01) and in place identity (*β_first_-β_repeat_* = −0.243, *p* < 0.05) between the two groups. Based on this, therefore, the results partially support hypothesis H7.

### Indirect effects test and multi-group analysis

According to the results in Table 4, the indirect effect of multisensory impressions on place dependence was significant through the emotional responses in both groups (first-timers: *β* = 0.474, *p* < 0.001; repeat tourists: *β* = 0.386, *p* < 0.001). Emotional responses partially mediate the relationship between multisensory impressions and place dependence across the two groups. Moreover, the difference that multisensory impressions had on place dependence through emotional responses between the two groups was significant (*β_first_-β_repeat_* = 0.088, *p* < 0.001). However, the indirect effect of multisensory impressions on place identity was not significant through emotional responses for either of the groups (first-timers: *β* = 0.017, *p* > 0.05; repeat tourists: *β* = 0.296, *p* > 0.05). There was no difference between first-time tourists and repeat tourists (*β_first_-β_repeat_* = −0.279, *p* > 0.05).

In addition, the indirect effect of multisensory impressions on place dependence was significant through cognitive responses for repeat tourists (*β* = 0.015, *p* < 0.01), but not for first-timers (*β* = 0.037, *p* > 0.05). Cognitive responses partially mediate the relationship between multisensory impressions and place dependence for repeat tourists. Moreover, the difference that multisensory impressions had on place dependence through cognitive responses between the two groups was significant (*β*_first_*-β*_repeat_ = 0.022, *p* < 0.05). However, in both groups, the indirect effect of multisensory impressions on place identity was significant through cognitive responses (first-timers: *β* = 0.313, *p* < 0.001; repeat tourists: *β* = 0.451, *p* < 0.001). Among the indirect effects of multisensory impressions on place identity, cognitive responses play a fully mediating role for first-time tourists. In contrast, cognitive responses play a partial mediating role for repeat tourists. Moreover, the difference that multisensory impressions had on place identity through cognitive responses between the two groups was significant (*β_first_-β_repeat_* = −0.138, *p* < 0.001). Based on this, the results also partially support hypothesis H7.

## Discussion and implications

### Discussion

This paper constructs a structural equation model to explore the mind–body mechanism of destination attachment across the two groups of first-time and repeat tourists, contributing to understanding the formation of destination attachment. The results show that physical factors (multisensory impressions) and psychological factors (emotional and cognitive responses) are important antecedents of destination attachment. The effects of the antecedents differ between first-time and repeat tourists. In addition, emotional responses and cognitive responses significantly mediate the relationship between multisensory impressions and destination attachment, which varies between first-time and repeat tourists. Further discussion of the results has yielded several interesting insights.

Specifically, multisensory impressions were found to be an antecedent of place identity only for repeat tourists, while cognitive responses were an antecedent of place dependence only for repeat tourists. However, emotional responses were not a prerequisite for place identity for either group. The possible reason for this is that forming the place identity requires a long period of contact. Repeat tourists with rich travel experiences will directly generate place identity under the stimulation of multisensory impressions. However, due to the lack of travel experience to the destination for first-time tourists, the simple sensory impression cannot directly activate place identity. It requires the intermediary of cognitive psychology to achieve this (Fuchs and Reichel, 2011). Compared with shallow emotional responses, tourists’ cognitive activities at the destination are usually accompanied by deep thinking and more complex psychological activities (Ayduk et al., 2002). Therefore, impulsive emotions cannot affect place identity. Once cognitive activities occur, place identity will first be affected, which has been confirmed in both first-time tourists and repeat tourists. Moreover, the depth of cognition will affect its spillover validity. Comparatively speaking, repeat tourists have higher cognitive responses, and their cognitive responses affect place identity and slightly affect place dependence. This also proves that, compared with place dependence, place identity has higher requirements on physical and mental factors (Bolam et al., 2006).

In addition, repeat tourists showed stronger relationships on multisensory impressions→cognitive responses (*β_first_* = 0.564 *< β_repeat_* = 0.711) and cognitive responses→place identity (*β_first_* = 0.312 *< β_repeat_* = 0.555) than did first-time tourists. However, first-time tourists showed stronger relationships on multisensory impressions→emotional responses (*β_first_* = 0.907 *> β_repeat_* = 0.609) and emotional responses→place dependence (*β_first_* = 0.586 *> β_repeat_* = 0.373) than did the repeat tourists. Therefore, the results highlight that multisensory impressions can positively affect emotional and cognitive responses, but the effects on first-time and repeat tourists differ. Further, first-time tourists tend to choose the marginal path to process information when receiving the same sensory stimuli, and the intuitive, emotional responses dominate, which, in turn, mainly affect place dependence (Jiang, 2020). On the other hand, repeat tourists tend to choose the central route to process information, and the cognitive response of rational thinking dominates, mainly affecting place identity.

Correspondingly, the indirect effect size of multisensory impressions on place dependence through emotional responses for first-time tourists was much larger than for repeat tourists (*β_first_ =* 0.474 *> β_repeat_* = 0.386). The indirect effect size of multisensory impressions on place identity through cognitive responses for repeat tourists was much larger than for first-time tourists (*β_first_ =* 0.313 < *β_repeat_* = 0.451). This result suggests that first-time tourists rely more on the mediating role of emotional responses than repeat tourists in the indirect effects of sensory impressions on destination attachment. In contrast, repeat tourists rely more on the mediating role of cognitive responses than first-time tourists.

Considering the size of indirect effects, multisensory impressions exert a more considerable impact on place dependence under the mediation of emotional processing. In contrast, multisensory impressions exert a more considerable impact on place identity under the mediation of cognitive processing. For physical factors, if they want to achieve a greater impact on destination attachment, they must go through emotional or cognitive psychological processes. In the physical and mental mechanism of destination attachment, psychological factors play a dominant role, while physical factors play a fundamental role in triggering the mechanism. Moreover, physical factors are important antecedents of psychological factors. Sensory impressions directly affect emotional and cognitive responses and can also indirectly affect cognitive responses through emotional responses and realize a chain-mediated mediation of destination attachment.

By further identifying the intermediary type, it is found that the emotional response partially mediates for first-time tourists. In contrast, the cognitive response plays the role of complete mediation. For repeat tourists, the emotional and cognitive responses play partial mediation roles. That is to say, with the increase in travel frequency, the body’s role becomes increasingly important in shaping the attachment to the destination.

By combining the weight changes of the second-order structure of multisensory impressions, emotional responses, and cognitive responses, it can be determined that visual (*β_first_* = 0.938) and auditory impressions (*β_first_* = 0.863) have strong explanatory power for first-time tourists. In contrast, taste (*β_repeat_* = 0.883) and tactile impressions (*β_repeat_* = 0.874) have strong explanatory power for repeat tourists. Positive surprise (*β_first_* = 0.978) has strong explanatory power for first-time tourists, while love (*β_repeat_* = 0.915) has strong explanatory power for repeat tourists. Novelty (*β_first_* = 0.919) has strong explanatory power for first-time tourists, while goal congruence (*β_repeat_* = 0.900) has strong explanatory power for repeat tourists. It can be speculated that, for first-time tourists, visual and auditory impressions directly or indirectly affect destination attachment mainly through positive surprise and novelty cognition. For repeat tourists, taste and tactile impressions directly or indirectly affect destination attachment, mainly through love and goal congruence.

### Theoretical implications

This paper proposes a people-body–mind-land framework of destination attachment formation, examining the body–mind utility effects therein. First, it verifies the effect of physical factors in the formation of destination attachment. The study found that while psychological factors play a leading role in shaping destination attachment, physical factors are the foundation. Although the direct effect of sensory impressions on destination attachment is not high, it can also indirectly influence destination attachment to a greater extent through the psychological mediating effects of emotion and cognition. As travel frequency increases, the body’s role becomes increasingly important. This affirms the value of the body in the formation mechanism of destination attachment and expands the applicability of the embodied theory in destination attachment research (Agapito et al., 2017).

Second, different from previous studies in which destination attachment was used as an overall construct (Skavronskaya et al., 2017), this study fine-grained the two-dimensional construct of destination attachment and examined the differences in the effects of mental factors on place dependence and place identity, respectively. Different psychological factors have different effects on different dimensions of destination attachment. Among them, place dependence is mainly driven by emotion. In contrast, place identity is mainly driven by cognition, which is consistent with the view posited by Backlund and Williams (2003) that the cognitive component is viewed as the place identity construct, and the emotional component is referred to as the place dependence construct. This study not only confirms the validity of the dual-system theory in explaining the psychology of destination attachment (Xu et al., 2019), but delineates different mechanisms of body–mind integration in motivating different attachment dimensions.

Finally, this study identifies differences in the body–mind mechanisms underlying destination attachment formation in first-time and repeat tourists. This study found that the physical and mental paths of first-time tourists are mainly as follows: multisensory impressions-emotional responses-place dependence, while the physical and mental paths of repeat tourists are multisensory impressions-cognitive responses-place identification. This means that the emotional response of first-time tourists is more prominent, while the cognitive response of repeat tourists is more prominent. This is in line with the position presented by Hwang et al. (2005) and Cao et al. (2021) to a certain extent: before the first-time tourists establish a connection with the destination, they mainly rely on the interaction between their senses and the physical environment to intuitively perceive and understand a destination. On the other hand, repeat tourists pay more attention to psychological meaning, and the level of place attachment is usually linked to the investment of cognitive resources. However, this may be different from the findings of Yolal et al. (2017) and Gursoy et al. (2014), who believe that first-time tourists place more importance on cognitive evaluation (service quality), while repeaters are loyal to their destination and rely more on emotional evaluation (satisfaction). The likely reason is that the context and destination of the studies are different, and the two studies ignore the underlying role of the body. Therefore, this study also provides a new perspective for exploring the differences in the travel behavior of first-time and repeat visitors from the perspective of body–mind integration.

### Managerial implications

This study provides a reference for coastal destination marketing organizations (DMOs) to cultivate tourists’ attachment to the destination from an embodied perspective. Firstly, destination marketers can design rich multisensory experiences for tourists to highlight the uniqueness of coastal destinations in an integrated way. The five dimensions (sight, hearing, taste, touch, and smell) of sensory experiences can be connected, targeted, and creatively based on the preference of different tourist markets. In this way, creating a sensory experience can better meet the heterogeneous needs of different tourists and promote the emotional connection between tourists and the destination. In particular, destination marketers need to focus on conveying the multisensory landscapes to tourists rather than waiting for this to be initiated by tourists. This allows the priming effect of sensory experiences on destination attachment to be maximized in accordance with the expectations of the DMO rather than being elusive.

Secondly, since the formation of destination attachment is fundamentally a body–mind integration process, destination marketers can design destination landscape presentations based on the connection ways of tourists’ bodies and mind to induce attachment. To make a few examples. By rendering the visual impact of blue skies and white clouds or enhancing the olfactory enjoyment of fresh air, a strong and intuitive aesthetic experience will induce tourists’ positive emotions of joy, love, and surprise, which is beneficial to place dependence. By delivering historical allusions to tourists through the way of scrolling on the electronic screen or situational interpretation, the cognitive thinking of tourists on the destination culture can be aroused. By providing some embodied entertainment activities, such as allowing tourists to fish and work together with local residents, or encouraging tourists to taste local delicacies, tourists will be stimulated to reflect on the unique local way of life and the value identification of the destination can be strengthened.

In addition, the different needs and function mechanisms of multisensory experiences of first-time and repeat tourists must be considered in targeted marketing.

Visual and auditory impressions are prone to evoking the psychological process of first-time tourists, while taste and tactile impressions likely stimulate the psychological process of repeat tourists. Once the multisensory impressions occur, the emotional responses of first-time tourists will primarily be triggered, which affects place dependence. However, the cognitive responses of repeat tourists will primarily be triggered, which affects place identity. Therefore, it is important to deliver landscape resources, iconic attractions, convenient tourism facilities, and other material satisfaction for first-time tourists to develop functional attachments. In contrast, for those who return, it is necessary to cultivate their interaction with the destination and strengthen their social relations at the destination to enable more in-depth sensory senses and cognitive psychological processes. This suggestion concurs with those of Li et al. (2008).

### Limitations and future research

This research has two limitations. First, this study only takes Hainan as a tourist destination as an example to verify the hypothesis model. The external validity of the conclusion requires further verification. In the future, the case location can be replaced, or a comparison of multiple coastal destinations can be conducted to investigate the mechanism of body–mind integration in the formation of destination attachment. Also, for a popular destination, there may always be a wide variety of tourism products, such as those that are religious, natural, or historical in nature. After tourists receive information through their senses, they will have different psychological preferences and reactions to various products. In future research, variables such as spiritual recovery, aesthetic perception, or cultural value can be added to the model to reflect the diversified reactions and refine the intermediary mechanism involved. The complex physical and mental mechanism of tourists’ destination attachment formation will be further explained.

## Data availability statement

The raw data supporting the conclusions of this article will be made available by the authors, without undue reservation.

## Author contributions

YD: conceptualization, methodology, project administration, and writing – original draft. YQ: formal analysis, reviewing and editing, and correspond with the journal. All authors contributed to the article and approved the submitted version.

## Conflict of interest

The authors declare that the research was conducted in the absence of any commercial or financial relationships that could be construed as a potential conflict of interest.

## Publisher’s note

All claims expressed in this article are solely those of the authors and do not necessarily represent those of their affiliated organizations, or those of the publisher, the editors and the reviewers. Any product that may be evaluated in this article, or claim that may be made by its manufacturer, is not guaranteed or endorsed by the publisher.
